# Comparison of the Accuracy of Four Malaria Diagnostic Methods in a High Transmission Setting in Coastal Cameroon

**DOI:** 10.1155/2019/1417967

**Published:** 2019-03-10

**Authors:** Marcel N. Moyeh, Innocent M. Ali, Dieudonné L. Njimoh, Akindeh M. Nji, Palmer M. Netongo, Marie S. Evehe, Barbara Atogho-Tiedeu, Stephen M. Ghogomu, Wilfred F. Mbacham

**Affiliations:** ^1^Department of Biochemistry and Molecular Biology, University of Buea, Buea, Cameroon; ^2^The Biotechnology Centre, University of Yaoundé 1, Yaoundé, Cameroon; ^3^The Biotechnology Unit, University of Buea, Cameroon; ^4^Department of Biochemistry, University of Dschang, Dschang, Cameroon; ^5^Department of Biochemistry, University of Yaoundé 1, Yaoundé, Cameroon

## Abstract

**Background:**

Despite recommendation from the World Health Organization that all malaria suspected patients undergo a parasitological confirmation using rapid diagnostic test or light microscopy prior to treatment, health facilities in remote malaria endemic settings sometimes resort to presumptive diagnosis of malaria for clinical management for various reasons. Following observation of this practice, we undertook a cross-sectional study aimed at comparing presumptive diagnosis based on axillary temperature, SD Bioline™ rapid test, and light microscopy as strategies for malaria diagnosis in the coastal region of Mutengene in the South West of Cameroon with the overall goal of supporting improved malaria diagnosis at local levels.

**Methodology:**

Venous blood from 320 participants was used to detect the presence of malaria parasite using SD Bioline™ mRDT and Giemsa stained microscopy or spotted on filter paper for PCR amplification of the 18s rRNA gene of* Plasmodium sp* following standard procedures. The axillary temperature of each participant was also measured. The sensitivity, specificity, and predictive values and their confidence intervals were determined for each of the methods with PCR as the reference. The area under the curve was used to estimate accuracy of diagnostic method and compared between test method using the X^2^ test with* P<0.05* considered significant.

**Results:**

The overall diagnostic sensitivities of presumptive diagnosis using axillary temperature, light microscopy, and SD Bioline™ were observed to be 74.30% (95%CI: 67.90-80.01), 94.86% (95%CI: 90.99-97.41), and 95.33% (95%CI: 91.57-97.74), respectively, and their respective diagnostic specificities were 53.77% (95%CI: 43.82-63.51), 94.34% (95%CI: 88.09-97.87), and 94.34%(95%CI: 88.09-97.89). SD Bioline™ had a diagnostic sensitivity of 91.80% [95%CI: 81.90-97.28] at a parasitaemia of less than 500 parasites/*μ*l of blood but a sensitivity of 100% for parasite counts above 500 parasites/*μ*l of blood. The predictive values of the positive test were highly comparable between light microscopy (90.09%, [95%CI: 83.61-94.18]) and SD Bioline™ mRDT (90.91%, [95%CI: 84.50-94.83]),* P=0.98* with kappa values of 0.898 but lower for presumptive diagnosis (50.89%, [95%CI: 43.72-58.03]), P<0.0001, and kappa value of 0.277. Perfect agreement was observed between SD Bioline™ mRDT and light microscopy (Cohen kappa= 0.924).

**Conclusions:**

The study showed that SD Bioline™ was as good as light microscopy in the diagnosis of malaria in remote areas of perennial transmission in South West Cameroon. This study equally revealed the limitations of presumptive diagnosis of malaria (as opposed to the use of RDTs or microscopy). Efforts should be made in such areas to promote parasitological confirmation of malaria using quality assured rapid tests or light microscopy for case management of malaria. The presence of nonnegligible levels of* Plasmodium ovale* in this study area indicate that treatment guidelines may require revision if same trend is proven in several other areas of same ecology.

## 1. Background

Although global reports show declining trends of malaria since 2010, the world malaria report of 2017 shows that the WHO African region still holds the record of 90% of malaria cases and deaths worldwide [1]. A total of 445000 deaths caused by malaria were recorded in 2016 and Cameroon alone accounted for 3% of this number [1]. The World Health Organization strongly advocates evidence-based treatment of malaria through demonstration of parasites or parasite parts/products in body analytes. Prompt and effective diagnosis of malaria through demonstration of the parasite and the infective species is essential in reducing morbidity and mortality as well as ultimate planning for the elimination of malaria in endemic settings.

Since the creation of the national malaria control program (NMCP) in 1998 and the reorganization of the National Roll Back Malaria Committee in 2002, the Cameroon malaria treatment and diagnostic guidelines have evolved enormously. While the level of endemicity determined the diagnostic method applied, the drug efficacy and resistance profile determined the national treatment recommended [2]. Malaria can be diagnosed presumptively using the signs and symptoms associated with it or through the demonstration of the parasite, its parts, or products in body fluids. Fever is the most prominent symptom of malaria which is often accompanied by chills, perspiration, anorexia, headaches, vomiting, and malaise. Residents of endemic areas are often familiar with these combinations of symptoms, and they frequently self-diagnose malaria based on symptoms alone [3]. In addition to these symptoms of uncomplicated malaria, other manifestations may develop that signal severe malaria which is almost always due to* Plasmodium falciparum*. It should be noted that these symptoms could also be manifestations of other febrile illnesses different from malaria making presumptive diagnosis a very subjective method of malaria diagnosis. Malaria can also be diagnosed microscopically by demonstrating the presence of parasites in Giemsa stained thick and thin films using the light microscope [4] or fluorescent stained blood samples observed using a fluorescent microscope [5]. Light microscopy can detect the presence of the parasite, the infecting species, and the parasite load but requires a trained microscope technician and a constant supply of power to run the equipment limiting the use of this method in resource poor settings. The infected parasite can also produce soluble antigens such as* Plasmodium falciparum* histidine rich protein 2 and 3 (PfHRP2/3), parasite lactate dehydrogenase (*pLDH*), and aldolase antigens that can be captured by monoclonal antibodies raised against these antigens [6]. This is the basis for the malaria rapid immune-chromatographic test commonly called rapid diagnostic tests (RDTs) used in malaria diagnosis. The immune-chromatographic methods have the advantage that they are easy to perform and do not require the services of a trained technician and electricity. Notwithstanding, the test method cannot be used to determine parasite load, the infecting species, and monitor treatment follow-up. Other methods of diagnosis include the use of Nested Polymerase Chain Reaction (PCR), Loop mediated isothermal amplification (LAMP), and nucleic acid sequence-based amplification (NABSA) techniques [7–10]. The Nested PCR method is a simple 2-round PCR that amplifies the 18s ribosomal RNA gene (using a thermocycler) which determines the presence of parasite as well as the infecting species [7] but cannot quantitate the parasite load. The LAMP and the NASBA techniques equally amplify, isothermally, the 18S ribosomal RNA gene and mRNA, respectively, but differ in that the NASBA can quantitate the parasite load using an in-vitro synthesized RNA competitor [9]

Before 2002, malaria diagnosis in Cameroon was mostly presumptive and light microscopy only used as a confirmation in cases of treatment failure and severe malaria [11]. By 2008, the national diagnostic policy was revised with presumptive diagnosis limited to children below five, pregnant women, and areas with limited access to light microscopy [11]. Despite this decision, access to universal diagnosis in the target group was limited given that light microscopy required a skilled technician to operate and a power source. Malaria rapid diagnostic test strips were adopted in 2009 and, after two years of resource mobilization, they were deployed in only 52 health districts nationwide [2]. At this time, the national malaria control program advocated the systematic diagnosis of all suspected malaria cases before the use of artemisinin-based combination therapy (ACT) in treatment [2].

Although several studies have been carried out to determine the accuracy of these RDTs with most using expert microscopy as the gold standard [12], inherent limitations of both light microscopy and RDTs [13] reduce reliability of such results. Therefore, a more sensitive and specific method is needed to assess the accuracies of these diagnostic methods and nested PCR has been shown to detect the presence of parasites below the detection limit of light microscopy and RDTs [7]. Also, reports from other areas suggest the emergence of parasite strains with a deletion of the* hrp2/3* gene implying the HRP2/3 based RDTs will not be very effective [14, 15]. Such parasite strains will test negative by* falciparum* specific RDTs in the presence of malaria parasites. It is therefore imperative that the diagnostic accuracy of these malaria RDTs be determined and compared with commonly used methods of diagnosis such as light microscopy and presumptive diagnosis. Effective diagnosis of malaria parasites in all suspected cases before treatment with ACTs would lead to a reduction in over prescription of the ACTs and reduce onset of resistance. This study was aimed at comparing the diagnostic accuracy of SD Bioline™ mRDT (both a histidine-based and a lactate dehydrogenase-based RDT) to that of light microscopy and presumptive diagnosis using PCR as the gold standard for the diagnosis of malaria in Mutengene, South West Region of Cameroon.

## 2. Methodology

### 2.1. Study Site/Population

The study was carried out within the context of a clinical trial between April and June 2013 in the Baptist Hospital Mutengene in the South West Region of Cameroon. Mutengene is found in the equatorial rain forest region along the Atlantic coastline where malaria transmission is recorded all year round [16]. The clinical trial was a phase III randomised controlled trial of artemisnin-based combination drugs aimed at evaluating the efficacy and safety of fixed dose combination of artesunate-amodiaquine (CoArsucam®) and dihydroartemisinin-piperaquine (Duo-cotecxin®) compared to artemether-Lumefantrine (CoArterm®) in the treatment of uncomplicated malaria. The results of the efficacy and safety trial have been published elsewhere [17]. The enrolled study participants included patients of both sexes aged 6 months and above visiting the outpatient department of the Baptist Hospital Mutengene. Participants with fever or a history of fever as well as participants without fever with other signs and symptoms suggestive of malaria were enrolled into the study. Samples/data were only collected from participants that consented to take part in the study. Patients with incomplete information were also excluded.

### 2.2. Sample/Data Collection and Malaria Diagnosis

Upon signing of the consent form, the axillary temperature of the participant and other vital signs and clinical symptoms were taken by a physician and entered in individual case report forms (CRF). Five millilitres of blood was collected from the enrolled participant by venepuncture using vacutainer needles and dispensed or stored in EDTA tubes. Thick and thin blood films were prepared (in duplicates), air dried, fixed (thin film only), and stained with Giemsa stain. The slides were observed at X1000 magnification under the light microscope and scored as negative or no parasites were seen after observing 200 high power fields. The slides were scored as positive if parasites were observed and the parasitaemia determined based on the assumption that there are 8000 white blood cells/*μ*l of blood [18].

A second laboratory technician performed the diagnosis using SD Bioline™ mRDT and this technician was blinded to the results of microscopy. With the help of blood transfer device supplied with the test cassettes, 5 *μ*l of blood was dispensed into the sample well and buffer added to the buffer well according to the manufacturer's instructions. The test was scored as positive if either of the PF or the Pan lines together with the control line were visible and negative if only the control line appeared. The intensities of the line colours on both the PF and* Pan* were scored as faint, bright, and very bright for the positive samples. The test was considered invalid when the control line was not visible and the test repeated. Test results for positive cases were handed over to the participants free of charge and referred to the physician for treatment as per national guidelines while further investigation into the cause of the febrile illness was undertaken for those with negative results.

Blood samples were equally spotted on labelled filter paper (Whatmann® No.3, Sigma-Aldrich, Germany), air dried, and stored in opaque envelopes alongside desiccant. These dot blood samples together with the second set of stained microscope slides were transported to the Laboratory of Cell and Molecular Biology of the University of Buea for molecular analysis. A second laboratory technician read and confirmed the results of microscopy and the opinion of a third technician was requested in case of discrepancies. DNA was extracted from the dried blood spots on filter paper by boiling in hot Chelex®-100 (Bio-Rad, Berkeley California, USA) as previously described [19]. A nested PCR amplification of the 18s ribosomal RNA gene using* Plasmodium* genus-specific primer and species-specific primers (Inqaba Biotec, Pretoria, South Africa) to detect the four species was carried out as already described [7]. The amplified PCR products were separated on a 2.5% Ethidium-bromide stained agarose gel alongside a 100 bp ladder (New Englands BioLabs, USA) and positive control DNA samples (MR4, Virginia, USA) with reference control DNA codes MRA-177, MRA-178, MRA-179, and MRA-180 for* P. falciparum, P. vivax, P. malariae*,* and P. vivax*,* respectively. *Negative controls constituted all the PCR components without the template DNA.

### 2.3. Statistical Analysis

Data from individual CRFs was double entered into excel and exported to MedCalc-version 8.0.0.1 for further analysis. The diagnostic sensitivity for each test method was considered the proportion of participants positive by test method compared to participants positive by PCR while the diagnostic specificity was considered the proportion of participants negative by test method compared to those negative by PCR. Positive Predictive Values (PPV) and Negative Predictive Values (NPV) were calculated as the proportion of true-positive results among all positively reacting samples and as the proportion of true negative results among all negatively reacting samples, respectively. The diagnostic accuracy of each test method was calculated with a 95% confidence interval and differences in proportion analysed by the chi-square test. The ability of each test to discriminate diseased from nondiseased cases and to compare diagnostic performance between test methods was evaluated using the receiver operating characteristics (ROC) curve analysis. Strength of agreement between test methods was also determined by estimating the Cohen's Kappa value and 95% CI. Statistical significance was set at 5%.

### 2.4. Ethical Consideration

This study obtained ethical approval from the Institutional Review Board of the Cameroon Baptist Convention Health Board (IRB2008-25 of 9 April, 2009), the WHO Ethics Review Committee decision (protocol MAL IRM 06 04) of 17 December, 2008, and the Institutional Review Board of the Biotechnology Centre of the University of Yaoundé I (002/BTC-IRB/UY1/2009). The trial was registered in the NIH trial registry (registration number: NCT01845701). The informed consent form was clearly written in English and given to participants to read and those who could not read, and the consent form was explained to them in Pidgin English. Consent to participate in the study for a minor was requested from the parents/legal guardians. The same procedure was used and data/samples were collected only from those that accepted and signed the consent form.

## 3. Results

**Figure 1:**
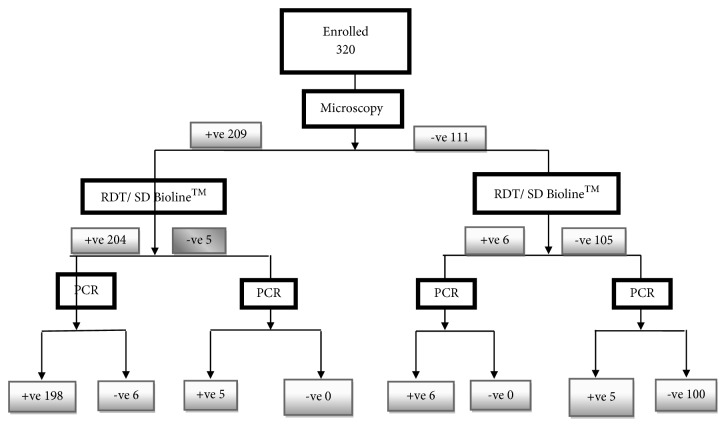
Flow chart for the diagnosis of malaria using regular light microscopy, SD Bioline™ mRDT and nested PCR as confirmatory method.

Three hundred and twenty subjects consented to the study, filled the study questionnaires, and provided blood samples used for the four malaria diagnostic methods herein evaluated. A total of 320 participants enrolled into the study comprising 43.2% males (138/320) and 56.9% females (56.9%). The ages of the participants ranged from 0.5 years to 65 years and a mean age of 17.8±14.9 years. The geometric mean parasite density GMPD was 5144 with parasitaemia ranging from 0 to 200.000 parasites/*μ*l of blood.  Figure 1 shows the detailed result of the trial profile of the 4 diagnostic methods. Screening through measurement of participant's axillary temperature showed that, out of the 320 participants enrolled, 65.0% (208/320) had fever (temperature ≥ 37.5°C) with a mean temperature of 38.0°C. In participants with fever at enrolment, 76.44% (159/208) had a positive test for malaria by PCR and 49.11% (55/112) of those that did not have fever had a positive test for malaria by PCR.

In blood samples monoinfected with* P. falciparum *alone, it was observed that as the parasite count increased the frequency of observing the Pan and PF line together increased.  Table 1 shows the proportion of samples presenting with either the PF line alone or PF and Pan lines at different parasite densities for samples positive for* P. falciparum *only.

The results presented in Table 1 showed that using SD Bioline™ mRDT samples with low levels of parasitaemia presented mostly the control and PF lines but as the parasite density gradually increased, the number of samples presenting with both the PF and the Pan lines also increased. Parasitaemia below 500 parasites/*μ*l had a high proportion of participants that presented only the control. As the parasite density increased the percentage proportion of participants with all three lines increased while that with just the control and PF lines decreased.

Nested PCR analysis of extracted DNA from all the samples indicated that 66.87% (214/320) of the samples were positive for malaria parasite. Eleven and six samples initially shown to be negative by light microscopy and SD Bioline™ mRDT, respectively, were positive for parasite DNA by nested PCR. Also, six samples negative by light microscopy and RDT were positive on amplification by PCR as shown in Figure 1 of the trial profile.

Table 2 presents the detailed results obtained for the diagnosis of malaria using light microscopy, SD Bioline™ mRDT, and PCR. The PCR diagnosis of malaria was considered the reference standard method of diagnosis in this study. Two samples which were initially negative by both light microscopy and SD Bioline™ mRDT were subsequently shown to be positive (*Plasmodium ovale)* by PCR species analysis while one negative by PCR was shown to be positive with a pan line by SD Bioline™ mRDT and was later genotyped to be* P. ovale. *Eight of the positive samples that presented with the Pan test line only were later identified as positive (*Plasmodium ovale) *by PCR analysis. Out of the 214 samples successfully amplified 82.71% (177/214) had monoinfection with* Plasmodium falciparum* and 5.14% (11/214) had monoinfection with Plasmodium ovale. Coinfections were seen with 4.67% (10/214) of the samples having a mixture of* ovale* and* falciparum* and 7.41% (16/216) having a mixture of falciparum and malariae. Monoinfection with* Plasmodium malariae* alone was not observed as well as cases of triple infection with* falciparum*,* ovale* and* malariae*. In all the samples analysed, no case of* Plasmodium vivax* malaria was seen. The overall disease prevalence using presumptive diagnosis, light microscopy, SD Bioline™ mRDT, and PCR was, respectively, 65%, 65.30%, 65.60%, and 66.90%. The nested PCR method showed positive for more samples than any other method of diagnosis.

Based on PCR as the “Gold standard” (see Table 3) the overall diagnostic sensitivities of presumptive diagnosis using axillary temperature, light microscopy, and SD Bioline™ mRDT were observed to be 74.30%, 94.86%, and 95.33%, respectively, and their respective diagnostic specificities were 53.77%, 94.34%, and 94.34%. SD Bioline™ mRDT had a diagnostic sensitivity of 91.80%  [95%CI: 81.90-97.28] at a parasitaemia of less than 500 parasites/*μ*l of blood but a sensitivity of 100% for parasite counts above 500 parasites/*μ*l of blood. Although the diagnostic sensitivities of light microscopy and SD Bioline™ mRDT were not significantly different from each other (P≫0.05), they were significantly higher than for presumptive diagnosis using Axillary temperature (P<0.0001). Plotting the ROC curve and performing ROC analysis, it was observed that light microscopy and SD Bioline™ mRDT had AUC (area under the ROC curve) values of 0.946 and 0.948% compared to 0.640 for presumptive diagnosis. This implied SD Bioline™ mRDT and light microcopy were relatively good in distinguishing diseased cases from nondiseased cases while presumptive diagnosis was very poor in differentiating diseased from nondiseased cases. Comparing presumptive diagnosis, SD Bioline™ mRDT, and light microscopy against PCR, we observed a Cohen kappa statistic of 0.277, 0.882, and 0.888, respectively. Light microscopy and SD Bioline™ mRDT had near perfect agreements with PCR (88.2% and 88.8%, respectively) while presumptive diagnosis had a poor agreement with PCR. When we considered light microscopy as the reference method, the AUC for SD Bioline™ mRDT was observed to increase to 96.1% with a Cohen kappa value of 92.4%. Comparing the area under the ROC curve between light microcopy and SD Bioline™ mRDT using PCR as reference method, it was observed that the difference was not significant (*p=0.82*) but highly significant when comparing SD Bioline™ mRDT or light Microscopy with presumptive diagnosis (p≪0.05).

The diagnostic sensitivity for the detection of* falciparum* malaria by SD Bioline™ mRDT was 95.51%  [95%CI: 91.34-98.04] and the diagnostic specificity, the PPV, and NPV for* falciparum* malaria alone were 95.24%  [95%CI: 89.24-98.44], 97.14%  [95%CI: 93.58-98.77], and 92.59%  [95%CI: 86.38-96.1], respectively. Using light microscopy as the “gold standard” test, the diagnostic sensitivity, specificity, PPV, and NPV of SD Bioline™ mRDT were observed to be 97.62%  [95%CI:94.53-99.22], 95.45%  [95%CI:89.71-98.51], 97.62%  [95%CI:94.57-98.98], and 95.45%  [95%CI:89.82-98.04], respectively.

## 4. Discussions

Highly sensitive diagnostic methods are important in endemic areas where the febrile illness can become rapidly fatal while high specificity is essential in all settings to reduce unnecessary treatment with antimalarial drugs thereby improving on diagnosis of other febrile illnesses. This study was designed to evaluate the diagnostic accuracy of rapid antigen detection test adopted by the national malaria control program for diagnosis of malaria in Cameroon and to compare this with conventional methods in use.

In this study, SD Bioline™ mRDT was unable to detect 5 samples microscopically confirmed positive in which parasite DNA was later detected by PCR. Such false negative results produced by SD Bioline™ mRDT could be accounted for by either intraspecies HRP2/HRP3 sequence variation in field isolates [20], deletions, or mutations of the* hrp-2/hrp3* such that the parasite no longer produces the antigen or produces a mutant antigen that is not recognized by antibodies on the test strip [14, 15]. Samples negative by microscopy but positive by SD Bioline™ RDT could represent samples collected from participants already on antimalarial drugs. Such samples will not have intact parasites but the gene product of* hrp2 *gene will still be in circulation. Such samples will test positive by SD Bioline™ RDT but microscopically they will show negative. Some studies have shown that HRP2 antigens could still remain in circulation for as long as 31 days following treatment [7, 21]. PCR identified 6 samples initially observed to be positive by light microscopy but negative by SD Bioline™ RDT. These might represent samples that fail to amplify the* hrp2/hrp3* gene fragment because the target sequence recognized by the oligonucleotide primers is deleted or mutated [14]. Six samples positive by RDT were also observed to be negative by PCR. In endemic areas, there may be no correlation between parasitaemia and antigenemia as some parasites are cleared very early in infection by the immune system leading to false positive RDT results or the parasites might be sequestered in deep capillaries of the liver, spleen, or bone marrow resulting in apparently false positive RDT results [22]. Such samples will be negative by PCR but positive by the rapid test method as circulating antigens will still be in blood samples.

The results showed that the diagnostic sensitivity of presumptive diagnosis of malaria was higher compared to results obtained by Batwala et al., [23] who carried out a similar study with* paracheck* in an area of similar climatic conditions and transmission intensity. The diagnostic specificity of presumptive diagnosis on the contrary was observed to be low implying there will be high chances of treating people who are not sick of malaria (false positives). Presumptive treatments based on axillary temperature remain challenging as the World Health Organization strongly recommends confirmation of parasites in body fluids for all suspected cases and treatment on the basis of clinical suspicion only considered when a parasitological diagnosis is not accessible [24]. Despite this high sensitivity of presumptive diagnosis, confident diagnosis of malaria requires a sensitivity of >90% [25]. This condition was not met by presumptive diagnosis using PCR as reference but by light microscopy and SD Bioline™ RDT. The results showed that the diagnostic sensitivities of SD Bioline™ RDT and light microscopy were not significantly different from each other (*P>0.05)*. The two test methods were not very much different from each other in their diagnostic performance with AUC >0.90 and perfect agreement between the test methods. The overall sensitivity of SD Bioline™ was higher than obtained for other forms of rapid diagnostic tests such as the* CareStart*™ Malaria* HRP*-2/*pLDH, *(Pf/pan)* Combo*, [26] and* OptiMAL* and* ICT* [27]. Despite inherent limitations posed by light microscopy [28], the diagnostic sensitivity was still high and compared to that of SD Bioline™ RDT. Using light microscopy as the reference diagnostic method, we observed that both methods could discriminate effectively between diseased and nondiseased cases with a Cohen kappa value of 0.924

Molecular analysis of* Plasmodium *species prevalence within the study area showed that most of the infections were due to monoinfection with* Plasmodium falciparum *(82.7%) and* Plasmodium Ovale *(5.1%).* Plasmodium falciparum *still remain economically the most important malaria causing parasite within the study area. The results are similar to those obtained in a study carried out by Bigoga et al. [16] in Tiko where 83.5% of malaria cases were caused by* falciparum *malaria. Contrary to results obtained by Cho-Fru et al. [29] that reported on the presence of substantial numbers of* Plasmodium vivax* cases in the same study area, no cases of* vivax *malaria were observed. The results differ from those obtained by Achonduh et al. [30] in Bangolan where a higher proportion of* Plasmodium malariae* was observed compared to* Plasmodium Ovale*. This shows an inverse relationship between the prevalence of* ovale* and* malariae* in the Guinea savanna region of Bangolan and the littoral forest region of Mutengene. While mixed infections of* Plasmodium falciparum* and* malariae* were the most common cause of malaria in Bangolan, monoinfection with* Plasmodium falciparum *was the main cause of malaria in the littoral forest region of Mutengene. The presence of* Plasmodium Ovale* in the study area necessitates the review of the current treatment protocol to cater for relapses common with* Plasmodium ovale* and* Plasmodium vivax* infections. The world health organization in its 2015 guidelines for malaria treatment recommends the addition of primaquine in the treatment protocol for patients with* ovale* and* vivax* infections so as to prevent relapses from hypnozoites, a phenomenon common with these species of* Plasmodium *[31]. In such cases, diagnosis of patients for glucose-6-phosphate dehydrogenase deficiency (G6PD) is essential. The substantial presence of* Plasmodium ovale *in the study area necessitates the inclusion of primaquine in treating non-*falciparum* malaria*. Plasmodium malariae *was found only coinfecting with* falciparum *malaria. Although* malariae *alone is a mild infection and never life-threatening, coinfecting with* falciparum *greatly changes the manifestation dynamics through nonspecific and cross-specific responses [32] that can lead to a nephrotic syndrome. Once established, this syndrome does not respond to treatment and carries a high rate of mortality [33].

## 5. Conclusions

The study showed that SD Bioline™ can be an addition to or alternative to light microscopy in the diagnosis of malaria in the South West Region of Cameroon. With a diagnostic sensitivity above 90% as required for confident diagnosis of malaria, SD Bioline™ could replace light microscopy in remote areas with difficult access to reagents and electricity. The study equally highlighted the inefficiency of presumptive diagnosis with sensitivity far below the recommendations for confident diagnosis of malaria. Efforts should therefore be made to replace such practices with parasitological confirmation of the parasite before ACT therapy. The presence of* Plasmodium ovale* in this study area requires a review of the current treatment protocol to cater for relapses.

## Figures and Tables

**Table 1:** Performance of SD Bioline™ mRDT Pf/Pan test lines with change is parasite density.

S/N	Parasite density (range)	Control	Control+ PF line	Control + PF+pan line	Total
1	NPS	95.37(103/108)	4.63 (5/108)	0.0 (0/108)	*108*

2	≤500	10.87 (5/46)	84.78 (39/46)	4.35 (2/46)	*46*

3	501-1000	0.0 (0/18)	44.44 (8/18)	55.56 (10/18)	*18*

4	1001-5000	0.0 (0/29)	41.38 (12/29)	58.62 (17/29)	*29*

5	5001-10000	0.0 (0/49)	16.67 (8/48)	83.33 (40/48)	*48*

6	>10000	0.0 (0/34)	5.88 (2/34)	94.12 (32/34)	*34*

*Total *		*108*	*74*	*101*	*283*

*Legend*. PF: histidine rich protein 2 line (HRP2 line), Pan: parasite specific lactate dehydrogenase line, NPS: No Parasites Seen. Numbers under Control, Control + PF line, and Control + PF + pan line indicate percentages and the numbers in bracket indicate number of observed cases in each parasitaemia range. Samples with mixed infections as well as samples with other forms of malaria were removed from this analysis.

**Table 2:** Microscopy and SD Bioline™ mRDT diagnosis results compared to nested PCR.

Microscopy		SD Bioline				Nested PCR				
		Neg	PF	Pan	PF/Pan	Neg	Pf	Pf/Pm	Po	Pf/Po
NPS	111	105	5	1	0	100	8	0	3	0

Positive	209	5	83	8	113	6	169	16	8	10

Total	320	110	88	9	113	106	177	16	11	10

*Legend*. NPS: No Parasite Seen, Neg: Negative, PF: HRP2 line, Pan: pLDH line, Pf: *Plasmodium falciparum*, Pm: *Plasmodium malariae*, and Po: *Plasmodium ovale*.

**Table 3:** Diagnostic sensitivity, specificity, PPV, NPV, and ROC analysis, and kappa values of agreement between test methods.

		Axillary temp (>37.5°C)	Light microscopy	SD Bioline™
Sensitivity	%	74.3	94.86	95.33
	(number/total)	159/214	203/214	204/214
	95%CI	67.90-80.01	90.99-97.41	91.57-97.74

Specificity	%	53.77	94.34	94.34
	(number/total)	57/106	100/106	100/106
	95%CI	43.82-63.51	88.09-97.86	88.09-97.89

NPV	%	50.89	90.09	90.91
	(number/total)	57/112	100/111	100/110
	95%CI	43.72-58.03	83.61-94.18	84.50-94.83

PPV	%	76.44	97.13	97.14
	(number/total)	159/208	203/209	204/210
	95%CI	72.26-80.17	93.96-98.66	93.98-98.67

ROC analysis	AUC	0.640	0.946	0.948
	Standard error	0.032	0.012	0.012
	95%CI	0.585-0.693	0.915-0.968	0.918-0.970

*Kappa values*		*0.277*	*0.882*	*0.888*

*Legend*. PPV: positive predictive value, NPV: negative predictive value, ROC: receiver operating characteristic curve, AUC: area under the ROC curve, RDT: rapid diagnostic test, and 95CI: 95% confidence interval. Nested PCR considered reference method.

## Data Availability

The data used to support the findings of this study are available from the corresponding author upon request.
